# multimark: an R package for analysis of capture–recapture data consisting of multiple “noninvasive” marks

**DOI:** 10.1002/ece3.1676

**Published:** 2015-10-13

**Authors:** Brett T. McClintock

**Affiliations:** ^1^ National Marine Mammal LaboratoryAlaska Fisheries Science CenterNOAA‐NMFS7600 Sand Point Way NESeattleWashington98115

**Keywords:** Bayesian multimodel inference, capture–recapture, Cormack–Jolly–Seber, latent multinomial, mark–recapture, Markov chain Monte Carlo, multiple lists, population size

## Abstract

I describe an open‐source R package, multimark, for estimation of survival and abundance from capture–mark–recapture data consisting of multiple “noninvasive” marks. Noninvasive marks include natural pelt or skin patterns, scars, and genetic markers that enable individual identification in lieu of physical capture. multimark provides a means for combining and jointly analyzing encounter histories from multiple noninvasive sources that otherwise cannot be reliably matched (e.g., left‐ and right‐sided photographs of bilaterally asymmetrical individuals). The package is currently capable of fitting open population Cormack–Jolly–Seber (CJS) and closed population abundance models with up to two mark types using Bayesian Markov chain Monte Carlo (MCMC) methods. multimark can also be used for Bayesian analyses of conventional capture–recapture data consisting of a single‐mark type. Some package features include (1) general model specification using formulas already familiar to most R users, (2) ability to include temporal, behavioral, age, cohort, and individual heterogeneity effects in detection and survival probabilities, (3) improved MCMC algorithm that is computationally faster and more efficient than previously proposed methods, (4) Bayesian multimodel inference using reversible jump MCMC, and (5) data simulation capabilities for power analyses and assessing model performance. I demonstrate use of multimark using left‐ and right‐sided encounter histories for bobcats (*Lynx rufus*) collected from remote single‐camera stations in southern California. In this example, there is evidence of a behavioral effect (i.e., trap “happy” response) that is otherwise indiscernible using conventional single‐sided analyses. The package will be most useful to ecologists seeking stronger inferences by combining different sources of mark–recapture data that are difficult (or impossible) to reliably reconcile, particularly with the sparse datasets typical of rare or elusive species for which noninvasive sampling techniques are most commonly employed. Addressing deficiencies in currently available software, multimark also provides a user‐friendly interface for performing Bayesian multimodel inference using capture–recapture data consisting of a single conventional mark or multiple noninvasive marks.

## Introduction

Capture–recapture methods historically relied on the physical capture, marking, and recapturing of animals for estimating population abundance and related demographic parameters such as survival (e.g., Williams et al. 2002). More recently, “noninvasive” capture–recapture sampling techniques are becoming commonplace for monitoring animal populations (e.g., Hammond 1990; Lukacs and Burnham 2005; O'Connell et al. 2010). Noninvasive marks can include natural pelt or skin patterns, scars, and genetic markers that enable individual identification in the absence of physical capture. Capture–recapture methods based on noninvasive marks have been applied to diverse taxa, including sharks (e.g., Holmberg et al. 2008), reptiles (e.g., Nair et al. 2012), ursids (e.g., Dreher et al. 2007), felids (e.g., Karanth and Nichols 1998; Ruell et al. 2009), and marine mammals (e.g., Hammond 1990; Wilson et al. 1999; Madon et al. 2011). While noninvasive capture–recapture methods have many advantages related to financial cost and animal welfare, they also pose new difficulties such as animal misidentification (Wright et al. 2009; Yoshizaki et al. 2009; Link et al. 2010; Morrison et al. 2011) and the complexity of multiple types of marks (Corkrey et al. 2008; Madon et al. 2011; Bonner and Holmberg 2013; McClintock et al. 2013).

Multiple marks can arise from sighting or camera surveys of species with natural mark patterns that are bilaterally asymmetrical (e.g., cetaceans, felids) or from multiple sources of noninvasive capture–recapture data being collected concurrently (e.g., fecal DNA sampling and visual surveys). With multiple marks, an encounter history is produced for each individual and mark type, but there is typically no reliable means to match them (unless each mark type is simultaneously observed at least once for every encountered individual). Because the number of unique individuals encountered must be known for standard capture–recapture analyses, the typical approach is to conduct separate analyses for each mark type and compare the results (e.g., Wilson et al. 1999; Berrow et al. 2012; Nair et al. 2012). However, given that sample sizes (and precision) may be considerably reduced, this is not as efficient as conducting an integrated analysis utilizing encounter histories arising from all mark types (McClintock et al. 2013). Additional costs of conducting separate analyses for each mark type include a limited ability to explore models with behavioral or cohort effects, and for capture–recapture models that condition on first encounter, a forfeiting of information from histories with the (apparent) first encounter occurring on the last sampling occasion. These limitations can be particularly important for the sparse datasets typical of rare and elusive populations for which noninvasive sampling techniques are most commonly employed.

Based on the latent multinomial model of Link et al. (2010), Bonner and Holmberg (2013) and McClintock et al. (2013) recently developed methods for performing integrated analyses of capture–recapture data consisting of multiple noninvasive marks. However, to my knowledge, their approaches have yet to be applied by practitioners. This is certainly not due to a lack of appropriate data (e.g., Wilson et al. 1999; Holmberg et al. 2008; Madon et al. 2011; Berrow et al. 2012; Nair et al. 2012) and is likely attributable to the mathematical and computational complexity of the models, as well as a lack of user‐friendly software for implementing them. Generalized software for performing Bayesian multimodel inference with capture–recapture data has also been lacking, thereby leaving these procedures largely inaccessible to nonstatisticians (e.g., Brooks et al. 2000; Durban and Elston 2005; King and Brooks 2008; Royle 2008; McClintock et al. 2013). These software needs were the motivation for multimark, an R (R Core Team 2013) package for Bayesian analysis of capture–recapture data consisting of multiple noninvasive marks.

After providing some additional background on capture–recapture with multiple marks, I briefly describe the models implemented in multimark. These currently include open population Cormack–Jolly–Seber (CJS) and closed population abundance models (e.g., Williams et al. 2002) with up to two mark types. Although originally motivated by the challenges posed by multiple noninvasive marks, multimark can also be used for analyses of conventional capture–recapture data consisting of a single‐mark type. Using real and simulated data for illustration, I provide an overview of the workflow for the package and a new analysis of left‐ and right‐sided encounter histories for bobcats (*Lynx rufus*) collected from remote single‐camera stations in southern California. Additional information, including help files, data, examples, and package usage, is available by downloading the multimark package from CRAN (http://cran.r-project.org) or github (https://github.com/bmcclintock/multimark). This article describes multimark version 1.3.0.

## Description

### Background

Capture–recapture data are typically represented by a collection of encounter histories **Y** = {**y**_1_, **y**_2_, …, **y**_*n*_}, where each element of **y**_*i*_ = (*y*_*i*,1_, *y*_*i*,2_, …, *y*_*i*,*T*_) indicates whether individual *i* was detected (*y*_*i*,*t*_ = 1) or not detected (*y*_*i*,*t*_ = 0) on each of *t* = 1,…,*T* sampling occasions. Typical analyses then proceed by formulating a likelihood conditional on the *n* unique individuals encountered (e.g., Williams et al. 2002). With two mark types, we instead have ${\tilde{Y}}_{m}=\left\{{\tilde{y}}_{m_{1}},{\tilde{y}}_{m_{2}},\ldots ,{\tilde{y}}_{m_{n_{m}}}\right\}$ for *m* ∈ {1,2}, where each element of ${\tilde{y}}_{m_{i}}=\left({\tilde{y}}_{m_{i,1}},{\tilde{y}}_{m_{i,2}},\ldots ,{\tilde{y}}_{m_{i,T}}\right)$ indicates individual *i* was detected $\left({\tilde{y}}_{m_{i,t}}=m\right)$ or not detected $\left({\tilde{y}}_{m_{i,t}}=0\right)$, and *n*_*m*_ is the number of unique individuals encountered for mark type *m*. We focus on situations where it is difficult (or impossible) to reliably match individuals from ${\tilde{Y}}_{1}$ and ${\tilde{Y}}_{2}$. In this case, although we know *n* ≤ *n*_1_+*n*_2_, *n* is nevertheless unknown and standard capture–recapture analysis methods cannot be reliably used for simultaneous inference using both sources of data.

Depending on the mark types and sampling design, it may sometimes be possible to observe both marks simultaneously within a sampling occasion. In this case, some of the encounter histories from ${\tilde{Y}}_{1}$ and ${\tilde{Y}}_{2}$ can be matched to unique individuals with certainty. For example, suppose images were collected during vessel‐based line transect surveys of surfacing whales, where mark type 1 corresponds to patch patterns on the left side and mark type 2 corresponds to patterns on the right side. If an individual happens to be photographed on both sides simultaneously on at least one sampling occasion, then the true encounter history for this individual would be known (i.e., left‐ and right‐sided images could be matched). This results in an additional set of *n*_known_ observed encounter histories, ${\tilde{Y}}_{\mathrm{known}}=\left\{{\tilde{y}}_{{\mathrm{known}}_{1}},{\tilde{y}}_{{\mathrm{known}}_{2}},\ldots ,{\tilde{y}}_{{\mathrm{known}}_{n_{\mathrm{known}}}}\right\}$, consisting of histories that are known with certainty (Table 1).

**Table 1:** Latent encounter histories **y** and the recorded histories $\left({\tilde{y}}_{1},{\tilde{y}}_{2},{\tilde{y}}_{\text{known}}\right)$ they generate for *T* = 2 sampling occasions and two mark types, where **y**=(*y*_1_,*y*_2_) for $y_{t}\in \left\{0,\;1,\;2,\;3,\;4\right\}$. Latent encounter histories are indexed by $j=1+\sum_{t=1}^{T}y_{t}5^{T-t}$, where the encounter types indicate nondetection (*y*_*t*_=0), type 1 encounter (*y*_*t*_=1), type 2 encounter (*y*_*t*_=2), nonsimultaneous type 1 and type 2 encounter (*y*_*t*_=3), and simultaneous type 1 and type 2 encounter (*y*_*t*_=4). If simultaneous encounters are possible, these results in some **y** being completely observable (as indicated by ${\tilde{y}}_{\text{known}}$)

*j*	**y**	${\tilde{y}}_{1}$	${\tilde{y}}_{2}$	${\tilde{y}}_{\text{known}}$
1	00	..	..	..
2	01	01	..	..
3	02	..	02	..
4	03	01	02	..
5	04	..	..	04
6	10	10	..	..
7	11	11	..	..
8	12	10	02	..
9	13	11	02	..
10	14	..	..	14
11	20	..	20	..
12	21	01	20	..
13	22	..	22	..
14	23	01	22	..
15	24	..	..	24
16	30	10	20	..
17	31	11	20	..
18	32	10	22	..
19	33	11	22	..
20	34	..	..	34
21	40	..	..	40
22	41	..	..	41
23	42	..	..	42
24	43	..	..	43
25	44	..	..	44

In essence, multimark facilitates the joint analysis of type 1 $\left({\tilde{Y}}_{1}\right)$, type 2 $\left({\tilde{Y}}_{2}\right)$, and known encounter histories $\left({\tilde{Y}}_{\mathrm{known}}\right)$, while accounting for uncertainty in the number of unique individuals encountered using extensions of the methodology proposed by Bonner and Holmberg (2013) and McClintock et al. (2013). While the mathematical and computational details are generally of little interest to ecologists, multimark performs these operations in the background and requires only simple data formatting and model specification formulas familiar to most R users.

### Models

multimark currently includes open population Cormack–Jolly–Seber (CJS) and closed population abundance models (e.g., Williams et al. 2002). These Bayesian implementations are similar in spirit to the CJS model of Royle (2008) and the abundance model of King et al. (2015). Given the latent encounter histories (**Y**) that generated the observed encounter histories $\left({\tilde{Y}}_{1},{\tilde{Y}}_{2},{\tilde{Y}}_{\text{known}}\right)$, the likelihood for the CJS model with two mark types is

(1) $$\left[Y|p,\delta ,\alpha ,\phi ,Q\right]\propto \prod_{i=1}^{n}\prod_{t=C_{i}+1}^{T}\pi_{i,t}$$

$$\pi_{i,t}=\left\{\begin{array}{cc} \left(1-p_{i,t}\right)\phi_{i,t-1}q_{i,t}+\left(1-\phi_{i,t-1}\right)\left(1-q_{i,t}\right) & \text{if}\;y_{i,t}=0\;\text{and}\;q_{i,t-1}=1\\ p_{i,t}\delta_{1}\phi_{i,t-1} & \text{if}\;y_{i,t}=1\\ p_{i,t}\delta_{2}\phi_{i,t-1} & \text{if}\;y_{i,t}=2\\ p_{i,t}\left(1-\delta_{1}-\delta_{2}\right)\left(1-\alpha \right)\phi_{i,t-1} & \text{if}\;y_{i,t}=3\\ p_{i,t}\left(1-\delta_{1}-\delta_{2}\right)\alpha \phi_{i,t-1} & \text{if}\;y_{i,t}=4\\ pc1 & \text{otherwise} \end{array}\right.$$

where *y*_*i*,*t*_=0 indicates a nondetection for individual *i* on occasion *t*,* y*_*i*,*t*_ = 1 indicates a type 1 encounter, *y*_*i*,*t*_ = 2 indicates a type 2 encounter, *y*_*i*,*t*_ = 3 indicates a nonsimultaneous type 1 and type 2 encounter, *y*_*i*,*t*_ = 4 indicates a simultaneous type 1 and type 2 encounter, $C_{i}\in \left\{1,\ldots ,T\right\}$ is the time of first capture for individual *i*,* p*_*i*,*t*_ is the detection probability for individual *i* during sampling occasion *t*,* δ*_*m*_ is the conditional probability of a type *m* encounter (given detection), *α* is the conditional probability of a simultaneous type 1 and type 2 encounter (given both mark types detected), *ϕ*_*i*,*t*−1_ is the survival probability between times *t*−1 and *t*, and *q*_*i*,*t*_ is an indicator for whether individual *i* was alive (*q*_*i*,*t*_ = 1) or not (*q*_*i*,*t*_ = 0) during sampling occasion *t*. For example, with *T* = 3, we, have cell probabilities $\pi_{i}\;=\;\prod_{t\;=\;C_{i}+1}^{T}\pi_{i,t}\;=\;\left(1-p_{i,3}\right)\phi_{i,2}q_{i,3}+\left(1-\phi_{i,2}\right)\left(1-q_{i,3}\right)$ for latent encounter history 020, *π*_*i*_ = *p*_*i*,2_ *δ*_1_ *ϕ*_*i*,1_ *p*_*i*,3_ *δ*_2_ *ϕ*_*i*,2_ for latent encounter history 412, *π*_*i*_ = (1−*p*_*i*,2_)*ϕ*_*i*,1_ *p*_*i*,3_(1−*δ*_1_−*δ*_2_)(1−*α*)*ϕ*_*i*,2_ for history 103, and *p*_*i*,3_(1−*δ*_1_−*δ*_2_)*αϕ*_*i*,2_ for history 034.

For added flexibility, *p* and *ϕ* are modeled using the probit link function:

$$\Phi \left(p_{i,t}\right)={x_{t}^{p}}^{\prime }\beta^{p}+z_{i}^{p}$$

$$\Phi \left(\phi_{i,t}\right)={x_{t}^{\phi }}^{\prime }\beta^{\phi }+z_{i}^{\phi }$$

where Φ() the cumulative distribution function of the standard normal density, $x_{t}^{p}$ and $x_{t}^{\phi }$ are row *t* of the design matrices for *p* and *ϕ*,***β*** ^*p*^ and ***β*** ^*ϕ*^ are the corresponding regression coefficients, and $z_{i}^{p}∼N\left(0,\sigma_{z^{p}}^{2}\right)$ and $z_{i}^{\phi }∼N\left(0,\sigma_{z^{\phi }}^{2}\right)$ are individual‐level effects that, respectively, allow for individual heterogeneity in detection and survival probability. Thus, while exploring the feasible set of latent encounter histories (**Y**), the parameters and latent variables to be estimated by multimark include ***β*** ^*p*^, ***β*** ^*ϕ*^, ***δ***,* α*,** Q**,** z** ^*p*^, **z** ^*ϕ*^, $\sigma_{z^{p}}^{2}$, and $\sigma_{z^{\phi }}^{2}$.

The probit link is implemented for CJS models in multimark because it facilitates a Gibbs sampler in the spirit of Albert and Chib (1993) and Laake et al. (2013). The probit link is very similar to the logit link, but the logit link has slightly fatter tails and is interpretable in terms of log‐odds. I note that this model reduces to that of Laake et al. (2013) for conventional capture–recapture data with a single‐mark type when *δ*_1_=1 and *δ*_2_=0 for $y_{i,t}\in \left\{0,1\right\}$.

Similarly, the likelihood for the closed population abundance model with two mark types is

(2) $$\left[Y|p,\delta ,\alpha ,N\right]\propto \frac{1}{{\left(p^{*}\right)}^{n}}\prod_{i=1}^{n}\prod_{t=1}^{T}\pi_{i,t}\times \text{Binomial}\;\left(n;N,p^{*}\right)$$

$$\pi_{i,t}=\left\{\begin{array}{cc} \left(1-p_{i,t}\right) & \text{if}\;y_{i,t}=0\\ p_{i,t}\delta_{1} & \text{if}\;y_{i,t}=1\\ p_{i,t}\delta_{2} & \text{if}\;y_{i,t}=2\\ p_{i,t}\left(1-\delta_{1}-\delta_{2}\right)\;\left(1-\alpha \right) & \text{if}\;y_{i,t}=3\\ p_{i,t}\left(1-\delta_{1}-\delta_{2}\right)\alpha & \text{if}\;y_{i,t}=4\\ 1 & \text{otherwise} \end{array}\right.$$

where *N* is the population size, and p* is the probability that a randomly selected individual is detected at least once. For example, returning to Table 1 with *T* = 2, we, for example, have cell probabilities $\pi_{i}\;=\;\prod_{t=1}^{T}\pi_{i,t}\;=\;\left(1-p_{i,1}\right)p_{i,2}\delta_{1}$ for latent encounter history 01, *π*_*i*_ = *p*_*i*,1_ *δ*_2_(1−*p*_*i*,2_) for history 20, *π*_*i*_ = *p*_*i*,1_ *δ*_2_ *p*_*i*,2_(1−*δ*_1_−*δ*_2_)(1−*α*) for history 23, and *π*_*i*_ = *p*_*i*,1_(1−*δ*_1_−*δ*_2_)*αp*_*i*,2_(1−*δ*_1_−*δ*_2_)(1−*α*) for history 43. As before, this model reduces to that for conventional capture–recapture data with a single‐mark type when *δ*_1_ = 1 and *δ*_2_ = 0 for $y_{i,t}\in \left\{0,1\right\}$.

For closed population models, *p* is modeled using the logit link function:

$$\text{logit}\left(p_{i,t}\right)={x_{t}^{p}}^{\prime }\beta^{p}+z_{i}^{p}$$

such that

$$p^{*}=1-\int_{-\infty }^{\infty }\prod_{t=1}^{T}\left(1-\frac{1}{1+\text{exp}(-\left({x_{t}^{p}}^{\prime }\beta^{p}+z^{p}\right))}\right)N\left(z^{p};0,\sigma_{z^{p}}^{2}\right)dz^{p}$$

is the probability of being detected at least once after accounting for individual heterogeneity in *p* (note that $p^{*}=1-\prod_{t=1}^{T}\left(1-{\text{logit}}^{-1}\left({x_{t}^{p}}^{\prime }\beta^{p}\right)\right)$ when $\sigma_{z^{p}}^{2}=0$). The parameters and latent variables to be estimated therefore include ***β*** ^*p*^, ***δ***,* α*,* N*,***z*** ^*p*^, and $\sigma_{z^{p}}^{2}$. Although a Gibbs sampler has been proposed for closed population models using the probit link and a complete data likelihood (McClintock et al. 2014), this does not apply to the “semicomplete” data likelihood in Eq. 2 (hence the traditional logit link is used). The primary utility of multimark is finding the set of latent encounter histories that are feasible given the observed encounter histories (sensu Link et al. 2010; Bonner and Holmberg 2013; McClintock et al. 2013, 2014). Given a feasible set of latent encounter histories (**Y**), fitting capture–recapture models such as Eqs. 1 or 2 is relatively straightforward.

## Workflow

### Multiple noninvasive marks

#### Data formatting

There are three types of multiple‐mark data that can be analyzed with multimark. These are the “never”, “sometimes”, and “always” data types, and they are named based on their respective probabilities of a simultaneous type 1 and type 2 encounter (Table 2). An example of the “never” data type is provided with multimark and includes 23 left‐sided $\left({\tilde{Y}}_{1}\right)$ and 23 right‐sided $\left({\tilde{Y}}_{2}\right)$ encounter histories for bobcats (*Lynx rufus*) collected from remote single‐camera stations in southern California over *T* = 8 sampling periods between July 2006 and January 2007 (McClintock et al. 2013; Alonso et al. 2015).

**Table 2:** Summary of three different types of multiple‐mark data. The data differ in terms of the latent encounter types (*y*_*t*_) that are possible based on the conditional probability of a simultaneous type 1 and type 2 encounter, *α* = Pr(*y*_*t*_ = 4¦*y*_*t*_ = 3 or *y*_*t*_ = 4)

Data type	*y*_*t*_	Constraints
“never”	{0, 1, 2, 3}	*α *= 0
“sometimes”	{0, 1, 2, 3, 4}	0 < *α* < 1
“always”	{0, 1, 2, 4}	*α* = 1

multimark expects observed encounter history data to be a matrix with rows corresponding to individuals and columns corresponding to sampling occasions. Because the bobcat data were collected from single‐camera stations, simultaneous left‐ and right‐sided encounters were not possible; hence, *α* = 0 and the rows consist of either 0's and 1's or 0's and 2's:



> library(multimark)
> data(bobcat)
> head(bobcat)




**Table:** 

	occ1	occ2	occ3	occ4	occ5	occ6	occ7	occ8
ID2	0	0	0	0	0	1	1	0
ID3	0	0	1	0	1	0	0	0
ID4	0	0	0	0	1	0	0	0
ID6	1	0	0	0	0	0	0	0
ID7	0	0	1	0	0	0	0	1
ID8	0	1	0	0	0	0	0	0



> tail(bobcat)




**Table:** 

	occ1	occ2	occ3	occ4	occ5	occ6	occ7	occ8
ID49	0	0	2	0	0	0	0	0
ID50	0	0	2	0	0	0	0	0
ID51	0	0	0	2	0	0	0	0
ID52	0	0	0	0	2	0	0	0
ID53	0	0	0	0	0	2	0	0
ID54	0	0	0	0	0	0	2	0

The ordering of the rows is unimportant; the package automatically recognizes which histories belong to ${\tilde{Y}}_{1}$, ${\tilde{Y}}_{2}$, and, if applicable, ${\tilde{Y}}_{\mathrm{known}}$.

The multimark function *processdata()* performs all additional data formatting. The basic inputs are the matrix of observed encounter histories (*Enc.Mat*) and the data type (*data.type*):



> bobcatsetup <− processdata(Enc.Mat=bobcat,data.type=“never”)




This creates an object of class *multimarksetup* that includes everything needed for model fitting and further analysis. In particular, *processdata()* calculates all of the necessary ingredients for identifying the feasible set of latent encounter histories (for technical details, see Bonner and Holmberg 2013; McClintock et al. 2013). There is also a feature enabling designation of individual encounter histories as known with certainty despite no simultaneous type 1 and type 2 detections (i.e., *y*_*i*,*t*_≠4 ∀ *t*), a situation that can arise from a previous physical capture or concurrent telemetry study (e.g., McClintock et al. 2013).

#### Model fitting

The package currently includes functions *multimarkCJS()* and *multimarkClosed()* for fitting CJS and closed population models, respectively, with two mark types. Use of these functions is perhaps best explained by example. To fit a simple closed population model assuming constant detection probability using the default settings:



> bobcat.dot <− multimarkClosed(mms=bobcatsetup,
mod.p= ^~^1)




Equivalently, *Enc.Mat* and *data.type* can be provided in lieu of the *mms* argument. In this case, *processdata()* is called from within *multimarkClosed()*:



> bobcat.dot <− multimarkClosed(Enc.Mat=bobcat,data.type=“never”,mod.p= ^~^1)




This creates a list, bobcat.dot, containing the MCMC output for the model (bobcat.dot$mcmc). The MCMC output is of class *mcmc*, which should be familiar to users of the R package coda (Plummer et al. 2006):



> summary(bobcat.dot$mcmc)
Iterations = 2001:12000
Thinning interval = 1
Number of chains = 1
Sample size per chain = 10000
1. Empirical mean and standard deviation for each variable, plus standard error of the mean:




**Table:** 

	Mean	SD	Naive SE	Timeseries SE
pbeta[(Intercept)]	−1.3302	0.23847	0.0023847	0.012847
N	35.6166	5.20282	0.0520282	0.277289
delta_1	0.3949	0.07296	0.0007296	0.007221
delta_2	0.4112	0.07269	0.0007269	0.006086




2. Quantiles for each variable:



**Table:** 

	2.5%	25%	50%	75%	97.5%
pbeta[(Intercept)]	−1.7987	−1.4982	−1.3330	−1.1614	−0.8783
N	28.0000	32.0000	35.0000	39.0000	48.0000
delta_1	0.2540	0.3444	0.3940	0.4457	0.5360
delta_2	0.2707	0.3605	0.4113	0.4611	0.5524



> coda::effectiveSize(bobcat.dot$mcmc)



**Table:** 

pbeta[(Intercept)]	N	delta_1	delta_2
344.5661	352.0563	102.0897	142.6469

Here, we can see posterior summaries for the default monitored parameters (*β* ^*p*^, *N*,* δ*_1_, *δ*_2_). Based on the effective sample sizes, it is clear that the default chain length is inadequate for this example; a typical “rule of thumb” is effective sample sizes >4000 for all quantities of interest.

Other common models for detection probability can be easily specified using linear model formulas for *mod.p*, including shorthands for time variation (*mod.p=˜time*), temporal trends (*mod.p=˜Time*), behavioral response to first capture (*mod.p=˜c*), and individual heterogeneity (*mod.p=˜h*). Additive or interaction terms can be included (e.g., *mod.p=˜time+c+h*,* mod.p=˜Time+I(Time^2)*, mod.p=˜time*c). User‐specified temporal covariates in detection probability can also be used:



> dummy <− rnorm(ncol(bobcat)) # some fake temporal covariates
> bobcatsetup <− processdata(Enc.Mat=bobcat,data.type=“never”,
covs=data.frame(cov1=dummy))
> bobcat.dummy_h <− multimarkClosed(mms=bobcatsetup,
mod.p=^∼^cov1+h,
parms=c(“pbeta”,“N”,“delta”,“sigma2_zp”))




The *covs* argument is a data frame used to enter discrete‐ or continuous‐valued temporal covariates, and *parms* specifies the parameters to monitor.

There are currently two options for specifying models for the conditional probabilities of type 1 and type 2 encounters (***δ***), the default *mod.delta=˜type* (i.e., *δ*_1_≠*δ*_2_), and *mod.delta=˜1* (i.e., *δ*_1_=*δ*_2_). The constraint *δ*_1_=*δ*_2_ will often be reasonable when type 1 and type 2 encounters arise from a very similar process, such as with left‐ and right‐sided images (see Example) . However, when type 1 and type 2 encounters arise from very different processes (e.g., fecal DNA and visual surveys), then specifying *δ*_1_≠*δ*_2_ is likely a model deserving consideration.

There are many additional arguments for specifying the number (*nchains*) and length (*iter*) of chains, including burn‐in and adaptive periods. For potential improvements in mixing, the number of “moves” used to update the feasible set of latent encounter histories at each iteration can be user specified (*maxnumbasis*; see Appendix S1). The default priors are “uninformative,” but user‐specified priors can be used for each parameter. Initial values can be automatically generated or user specified for each parameter.

The function *multimarkCJS()* works in exactly the same fashion, with the only notable difference being specification of models for *ϕ* (in addition to *p* and ***δ***). Although CJS‐specific data are not included with multimark, data can be simulated using the *simdataCJS()* function (or *simdataClosed()* for closed populations):



> CJSdata <‐ simdataCJS(N=100,noccas=7,pbeta=−0.25,phibeta=1,delta_1=0.2,
delta_2=0.5,alpha=0.5,sigma2_zphi=0.25,data.type=“sometimes”)
> Enc.Mat<− CJSdata$Enc.Mat
> head(Enc.Mat)




**Table:** 

	[,1]	[,2]	[,3]	[,4]	[,5]	[,6]	[,7]
[1,]	1	0	0	0	0	0	0
[2,]	1	0	0	0	0	0	0
[3,]	0	0	1	0	0	0	0
[4,]	2	0	2	3	4	0	2
[5,]	4	1	0	0	0	0	0
[6,]	4	3	0	0	0	0	0



> CJSsetup <‐ processdata(Enc.Mat=Enc.Mat,data.type=“sometimes”)
> CJS.dot.h <‐ multimarkCJS(mms=CJSsetup,
mod.p=^∼^1,mod.delta=^∼^type,mod.phi=~h,
parms=c(“pbeta”,“delta”,“alpha”,“phibeta”,“sigma2_zphi”),
nchains=2,iter=45000,burnin=5000) > summary(CJS.dot.h$mcmc)






Iterations = 5001:45000
Thinning interval = 1
Number of chains = 2
Sample size per chain = 40000
1. Empirical mean and standard deviation for each variable, plus standard error of the mean:




**Table:** 

	Mean	SD	Naive SE	Timeseries SE
pbeta[(Intercept)]	−0.23929	0.12483	0.0004413	0.0034456
phibeta[(Intercept)]	1.39411	0.30023	0.0010615	0.0123494
alpha	0.51730	0.11716	0.0004142	0.0026694
sigma2_zphi	0.04021	0.09187	0.0003248	0.0039210
delta_1	0.20576	0.04557	0.0001611	0.0010992
delta_2	0.59324	0.05317	0.0001880	0.0009821



2. Quantiles for each variable:




**Table:** 

	2.5%	25%	50%	75%	97.5%
pbeta[(Intercept)]	−0.471868	−0.326159	−0.24406	−0.15725	0.01808
phibeta[(Intercept)]	0.916923	1.186706	1.35644	1.55790	2.08917
alpha	0.295278	0.434658	0.51570	0.59789	0.74769
sigma2_zphi	0.002618	0.007179	0.01413	0.03391	0.26382
delta_1	0.122343	0.173677	0.20383	0.23541	0.30069
delta_2	0.486954	0.557618	0.59420	0.62968	0.69478

An additional feature for *multimarkCJS()* is simple specification of “age” and cohort effects for *p* (*mod.p=˜age* and *mod.p=˜cohort*) and *ϕ* (*mod.phi=˜age* and *mod.phi=˜cohort*), which can be useful for investigating structure related to time since first capture and time of initial capture, respectively. These variables by default include a level for each unique age or cohort, but they can be binned to reduce the number of levels using additional arguments.

### Single‐mark type

For conventional capture–recapture data consisting of a single‐mark type, encounter histories are formatted the same way but now consist solely of 1's (detections) and 0's (nondetections). The package currently includes the functions *markCJS()* and *markClosed()* for fitting conventional CJS and closed population models, respectively. These functions are essentially wrappers that “trick” *multimarkCJS()* and *multimarkClosed()* to fit models with a single‐mark type. The functions *simdataCJS()* and *simdataClosed()* can also be used to simulate encounter history data with a single‐mark type by setting the arguments *delta_1=1* and *delta_2=0*. For example, to simulate CJS data and fit a model with constant detection probability and individual heterogeneity in survival:



> singleCJSdata <‐ simdataCJS(delta_1=1,delta_2=0,
pbeta=−0.25,phibeta=1,sigma2_zphi=0.25)
> Enc.Mat <‐ singleCJSdata$Enc.Mat
> singleCJS.dot.h <‐ markCJS(Enc.Mat=Enc.Mat,mod.p=^∼^1,mod.phi=^∼^h,
parms=c(“pbeta”,“phibeta”,“sigma2_zphi”),
nchains=2,iter=45000,burnin=5000)




There are fewer arguments for *markCJS()* and *markClosed()* because there is only one mark type (e.g., the arguments *mms* and *mod.delta* are no longer necessary), but the remaining arguments are specified exactly as for *multimarkCJS()* and *multimarkClosed()*.

### Further analysis

While the coda package can be used to summarize, plot, and assess convergence of MCMC samples from *markClosed()*,* multimarkClosed()*,* markCJS()*, and *multimarkCJS()*, several additional functions are available for further analysis of model output. Because link functions are used for *p* and *ϕ*, the functions *getprobsClosed()* and *getprobsCJS()* provide estimates on the real scale. For example, we can compare the probabilities of capture (*p*) and recapture (*c*) when there is a behavioral response to first capture (i.e., *mod.p*=˜*c*):



> bobcat.c <− multimarkClosed(mms=bobcatsetup,mod.p=^∼^c)
> pc <− getprobsClosed(bobcat.c)
> summary(pc[,c("p1]","c[2]")])
Iterations = 2001:12000
Thinning interval = 1
Number of chains = 1
Sample size per chain = 10000
1. Empirical mean and standard deviation for each variable, plus standard error of the mean:




**Table:** 

	Mean	SD	Naive SE	Timeseries SE
p[1]	0.137	0.05407	0.0005407	0.005286
c[2]	0.259	0.05148	0.0005148	0.002981



2. Quantiles for each variable:



**Table:** 

	2.5%	25%	50%	75%	97.5%
p[1]	0.0485	0.09735	0.1321	0.1716	0.2538
c[2]	0.1619	0.22273	0.2569	0.2931	0.3639

Here, p[1] and c[2] refer to the probabilities of capture and recapture at times *t* = 1 and *t* = 2, respectively.

Based on Barker and Link (2013), Bayesian multimodel inference using reversible jump MCMC is implemented through the functions *markClosed()*,* multimarkClosed()*,* markCJS()*, and *multimarkCJS()*. Using this approach, models are first run individually and a Gibbs sampler explores the model space using the individual model MCMC output. All that must be provided to the multimodel inference functions is a list containing the output from at least two models. The models must have the same number and length of MCMC chains, and all model parameters must be monitored (this is accomplished by setting *parms=“all”*):



> bobcat.dot <‐ multimarkClosed(mms=bobcatsetup,mod.p=^∼^1,parms="all")
> bobcat.c <‐ multimarkClosed(mms=bobcatsetup,mod.p=^∼^c,parms="all")
> bobcat.time <‐ multimarkClosed(mms=bobcatsetup,mod.p=^∼^time,parms="all")
> bobcat.h <‐ multimarkClosed(mms=bobcatsetup,mod.p=^∼^h,parms="all")
> modlist <‐ list(mod1=bobcat.dot,mod2=bobcat.c,
mod3=bobcat.time,mod4=bobcat.h)
> bobcat.M <‐ multimodelClosed(modlist=modlist)




The list bobcat.M includes RJMCMC output (bobcat.M$rjmcmc) for parameters common to all models (which can be specified using the argument *monparms*) and posterior model probabilities (bobcat.M$pos.prob). Other arguments for *multimodelClosed()* and *multimodelCJS()* include prior model probabilities (*modprior*) and user‐specified proposal distributions for moving between models.

## Example

I will now provide results from a new closed population analysis of the bobcat data performed in multimark. Previous analyses of these data include McClintock et al. (2013), who performed an integrated analysis but for a limited model set that did not include behavioral or individual effects, and Alonso et al. (2015), who performed standard single‐sided analyses that could not investigate behavioral responses to first capture. Using multimark, it is possible to conduct a more complete analysis using both left‐ and right‐sided encounter histories that includes no effects, temporal effects, behavioral effects, and individual effects in detection probability. I also investigated two models for ***δ*** (*δ*_1_≠*δ*_2_ and *δ*_1_=*δ*_2_) because it is reasonable to suspect that the conditional probabilities of left‐sided (type 1) and right‐sided (type 2) encounters are similar.

Fitting all possible additive combinations yielded 16 models using the default “noninformative” priors for *multimarkClosed()*:

$$\begin{array}{cc} \beta^{p}∼ & N(0,1.75)\\ \delta ∼ & \left\{\begin{array}{cc} \text{Beta}\;(1,1) & \text{if}\;\delta_{1}=\delta_{2}\\ \text{Dirichlet}\;(1,1,1) & \text{if}\;\delta_{1}\neq \delta_{2} \end{array}\right.\\ z_{i}^{p}∼ & N(0,\sigma_{z^{p}}^{2})\\ \sigma_{z^{p}}∼ & \text{half‐Cauchy}\;(25)\\ N\propto & \frac{1}{N} \end{array}$$

With two chains each consisting of two million iterations (with thinning every 20 iterations to reduce memory requirements), the simplest models required 12 min on a computer running Windows 7 (3.4 GHz Intel Core i7 [Intel Corporation, Santa Clara, CA], 16 GB RAM), while the more complicated models including time variation required at most 2 h. These relatively fast run times are attributable to multimark's parallel processing of MCMC algorithms written in the C programming language (Kernighan and Ritchie 1988). Bayesian multimodel inference was performed with *multimodelClosed()* using the default equal prior model weights, where 300000 iterations for each chain required 2.6 h. The longer run time for *multimodelClosed()* owes to the number of models and the RJMCMC algorithm being written entirely in R.

Models including a positive behavioral response to first capture accounted for 0.51 of the posterior model weight, while models including *δ*_1_=*δ*_2_ accounted for 0.78 of posterior model weight (Table 3). Model‐averaged posterior modes were *N* = 35 (highest posterior density interval: 26–101; Fig. 1) for population abundance, *p* = 0.15 (HPDI: 0.04–0.27) for capture probability, and *c* = 0.21 (HPDI: 0.07–0.33) for recapture probability. With *δ*_1_=*δ*_2_=0.41 (HPDI: 0.30–0.50) based on the model with the highest posterior probability, both‐sided encounters were relatively infrequent for these data (1−*δ*_1_−*δ*_2_=0.18; HPDI: 0.00–0.39).

**Figure 1:**
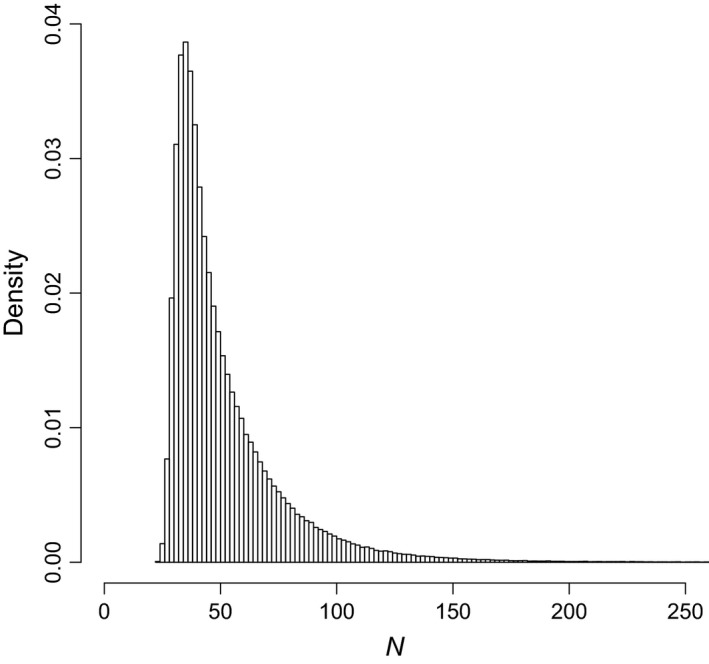
Model‐averaged posterior distribution of population abundance (*N*) for the bobcat data.

**Table 3:** Posterior model probabilities (PMM) and abundance estimates for the bobcat data. Summaries include posterior modes (*N*), 95% highest posterior density intervals (HPDI), effective sample sizes (ESS), and Gelman–Rubin–Brooks diagnostics (GRB) for *N*. Models for detection probability (p) included no effects (˜1), behavioral effects (˜c), time effects (˜time), and individual effects (˜h). Models for the conditional probability of a left‐ or right‐sided encounter (delta) included *δ*_1_=*δ*_2_ (˜1) and *δ*_1_≠*δ*_2_ (˜type)

Model	PMM	*N*	HPDI	ESS	GRB
p(˜c)delta(˜1)	0.30	38	27–91	38944	1.00
p(˜1)delta(˜1)	0.22	33	26–46	54696	1.00
p(˜h)delta(˜1)	0.16	46	29–114	11685	1.00
p(˜c + h)delta(˜1)	0.09	50	29–145	18544	1.00
p(˜c)delta(˜type)	0.09	38	27–90	35054	1.00
p(˜1)delta(˜type)	0.06	33	26–46	53961	1.00
p(˜h)delta(˜type)	0.05	48	29–113	12099	1.00
p(˜c + h)delta(˜type)	0.03	51	29–146	17276	1.00
p(˜time + h)delta(˜1)	0.00	47	28–115	14414	1.00
p(˜c + time + h)delta(˜1)	0.00	45	28–116	21473	1.00
p(˜time)delta(˜1)	0.00	33	26–45	47781	1.00
p(˜c + time)delta(˜1)	0.00	33	25–78	35169	1.00
p(˜time + h)delta(˜type)	0.00	50	29–118	13882	1.00
p(˜c + time + h)delta(˜type)	0.00	46	27–115	21337	1.00
p(˜time)delta(˜type)	0.00	33	26–45	49425	1.00
p(˜c + time)delta(˜type)	0.00	32	25–78	35360	1.00

For comparison, I performed conventional left‐ and right‐sided analyses for these data using *markClosed()* and *multimodelClosed()*. Because models for ***δ*** and behavioral response do not apply, the candidate model set was limited to *mod.p=˜1*,* mod.p=˜time*,* mod.p=˜h*, and *mod.p=˜time+h* for these single‐sided analyses. As before, the default “noninformative” priors were used, and the length and number of chains, burn‐in periods, and adaptive periods were also the same. For the left‐side analysis, the constant detection probability model accounted for 0.95 of the posterior model weight, while the individual heterogeneity model accounted for 0.04 of posterior model weight. Model‐averaged posterior modes were *N*=32 (HPDI: 24–52) for population abundance and *p*=0.12 (HPDI: 0.07–0.19) for capture probability. For the right‐side analysis, the constant detection probability model accounted for 0.6 of the posterior model weight and the individual heterogeneity model accounted for 0.39 of posterior model weight. Model‐averaged posterior modes were *N*=33 (HPDI: 23–85) for population abundance and *p*=0.12 (HPDI: 0.04–0.19) for capture probability. These conflicting results demonstrate the unenviable position one can often find oneself when conducting separate analyses for different mark types from the same population. One may be tempted to choose the “most precise” estimate based on the left‐side analysis, but the integrated analysis suggests this would considerably underestimate the uncertainty about *N*. Choosing the “more conservative” right‐sided results or averaging the *N* estimates from the left‐ and right‐sided analyses would also underestimate the uncertainty about *N* based on the integrated analysis. This discrepancy is likely attributable to the potential behavioral response to first capture identified by the integrated analysis.

## Discussion

I have described some of the key features of multimark, a new R package for the analysis of capture–recapture data consisting of a single conventional mark or multiple noninvasive marks. The package currently includes open population CJS and closed population models, with functions for derived parameters (e.g., *ϕ*,* p*) and multimodel inference. It adds to the growing toolbox of freely available software for the analysis of nonspatial (e.g., White and Burnham 1999; Choquet et al. 2009; Laake et al. 2013; Laake 2013) and spatial (e.g., Gopalaswamy et al. 2012; Efford 2015) capture–recapture data, but it is the first to combine otherwise irreconcilable encounter histories arising from multiple‐mark types. Although initially developed for integrated analyses of left‐ and right‐sided images for bilaterally asymmetrical species, the package can be used to jointly analyze data arising from any two types of marks. For example, multimark could be used to integrate an analysis of encounter histories arising from genetic (e.g., hair or fecal) and visual (e.g., photograph ID) detections (sensu Madon et al. 2011; but see Bonner 2013). multimark is also the first capture–recapture software to implement generalized Bayesian multimodel inference based on the RJMCMC algorithm proposed by Barker and Link (2013).

Relative to previous applications using multiple marks (Bonner and Holmberg 2013; McClintock et al. 2013), the relatively fast computation times of the package are attributable to its use of “semicomplete” data likelihoods (King et al. 2015), parallel processing, and MCMC algorithms written in C (instead of R). Because parallel processing relies on the parallel package (R Core Team 2013), first‐time Windows and OS X users can expect a firewall pop‐up dialog box asking if an R process should accept incoming connections. Memory requirements are minimized by conditioning on the observed encounter histories when identifying the feasible set of latent encounter histories. To facilitate better mixing, multimark improves the MCMC algorithms proposed by Bonner and Holmberg (2013) and McClintock et al. (2013, 2014) by avoiding latent encounter history proposals with negative frequencies in a manner that requires no proposal tuning (see Appendix S1 for details).

Many potentially desirable extensions to multimark are possible. These include a broader suite of capture–recapture models, such as multistate and robust design models (e.g., Williams et al. 2002). In addition to individual‐level heterogeneity, “random effect” distributions for temporal or user‐specified covariates could also be incorporated (e.g., Laake et al. 2013). More general modeling formulae for ***δ*** and *α* would allow additional hypotheses related to detection to be explored. The package could also be extended to accommodate >2 mark types and additional link functions. Although many individual covariates tend to be difficult (or impossible) to observe with noninvasive sampling, some (e.g., sex) may be easily discernible for each mark type. For these cases, it would be relatively straightforward to extend multimark to accommodate individual covariates. Other extensions include spatially explicit models (e.g., Royle 2015) and allowing for partial overlap in the sampling periods for each mark type. Practitioners interested in such extensions are encouraged to contact the author.

## Conflict of Interest

None declared.

## Supporting information


**Appendix S1.** Updating of latent encounter history frequencies in multimark
