# Supplementary material for: multimark: an R package for analysis of capture–recapture data consisting of multiple “noninvasive” marks
Source: Ecol Evol. 2015 Oct 13;5(21):4920–31. doi: 10.1002/ece3.1676 (PMC4662319; doi:10.1002/ece3.1676)
Supplement: Supplementary file 1 — Appendix S1. Updating of latent encounter history frequencies in multimark [file ECE3-5-4920-s001.pdf]

Appendix S1. Updating of latent encounter history frequencies in `multimark`

Brett T. McClintock[1]

National Marine Mammal Laboratory

Alaska Fisheries Science Center
NOAA National Marine Fisheries Service
Seattle, Washington, U.S.A.
[1]*Email:* brett.mcclintock@noaa.gov

July 31, 2015

The feasible set of latent encounter histories $(\mathbf{Y})$ is explored in `multimark` using an extension of the MCMC algorithms proposed by Bonner & Holmberg (2013) and McClintock *et al.* (2013, 2014) that were originally conceived for a different application by Link *et al.* (2010). The new algorithm conditions on the observed data (thus reducing the dimension of the problem) and only proposes updates with non-negative latent encounter history frequencies, $\mathbf{x} = (x_1, x_2, \ldots, x_{5^T})$. Let $\mathbf{r}$ denote the set of $4^T - 2^{T+1} + 1$ indices for latent encounter histories that spawn >1 observed history, and let $x_{j(1)}$ and $x_{j(2)}$ denote the corresponding frequencies for type 1 and type 2 histories that arise from latent encounter history $j \in \mathbf{r}$. Referring back to Table 2 with $T = 2$, $\mathbf{r} = \{4, 8, 9, 12, 14, 16, 17, 18, 19\}$ and one potential update would involve frequencies for latent history '31' (x_{17}) and its progeny '11' $(x_{17(1)} = x_7)$ and '20' $(x_{17(2)} = x_{11})$.

When conditioning on the observed encounter histories, the size of the problem is typically greatly reduced because many of the potential latent histories and corresponding moves are not permissable. For example, with $T = 2$, if encounter history '11' was never observed, then $x_7 = x_9 = x_{17} = x_{19} = 0$ can be ignored and $j \in \{9, 17, 19\}$ can be removed from $\mathbf{r}$ for subsequent computations.

Starting from a permissible $\mathbf{x}$ conditional on the observed encounter histories, the algorithm proceeds as follows:

1. Randomly draw a latent encounter history index $r \in \mathbf{r}^*$, where $\mathbf{r}^*$ is the subset of $\mathbf{r}$ with corresponding frequencies that satisfy $\min(x_1, x_j) + \min(x_{j(1)}, x_{j(2)}) > 0$ for $j \in \mathbf{r}$.

2. Randomly draw c_r from the integer set $\{-\min(x_1, x_r), \ldots, -1, 1, \ldots, \min(x_{r(1)}, x_{r(2)})\}$.

3. Propose $x^*_r = x_r + c_r$, $x^*_{r(1)} = x_{r(1)} - c_r$, and $x^*_{r(2)} = x_{r(2)} - c_r$.

4. Apportion $\mathbf{x}^*$ to individuals following McClintock *et al.* (2014), and accept proposed move based on the Metropolis-Hastings ratio described therein [pp. 2470-2472, steps 9(b)-9(c)].

Any additional constraints, such as those resulting from encounter histories being designated as known with certainty using the *known* argument in *processdata()*, are accounted for by simple modifications to steps 1-2. In terms of mixing, it can sometimes be advantageous to explore more than one move at a time. At each iteration of the chain, the argument *maxnumbasis* specifies how many times to perform steps 1-3 in sequence before evaluating step 4. The default for *multimarkCJS()* and *multimarkClosed()* is *maxnumbasis=1*.

Note that because `multimark` uses "semi-complete" data likelihoods that condition on the number of unique individuals encountered at least once (n), the dimension of the data-augmented encounter histories (M) described in McClintock *et al.* (2014) is determined by the number of observed encounter histories (i.e. $M = n_1 + n_2 + n_{known}$) such that $x_1 = M - n$. Letting $w_i \sim \text{Bernoulli}(\psi)$ be an indicator for whether or not individual i belongs to the n unique individuals encountered at least once (i.e. $\sum_{i=1}^{M} w_i = n$), then $w_i = 1$ if $H_i > 1$ (otherwise $w_i = 0$), where H_i is the latent encounter history index for individual i $\left(\sum_{i=1}^{M} I(H_i = j) = x_j\right)$, and $\psi \sim \text{Beta}\left(a_\psi^0, b_\psi^0\right)$ is the probability that a randomly selected individual from the M observed individuals belongs to the n unique individuals encountered at least once. The defaults in *multimarkCJS()* and *multimarkClosed()* are $a_\psi^0 = b_\psi^0 = 1$.

References

Bonner, S.J. & Holmberg, J. (2013) Mark-recapture with multiple, non-invasive marks. *Biometrics*, **69**, 766–775.

Link, W.A., Yoshizaki, J., Bailey, L.L. & Pollock, K.H. (2010) Uncovering a latent multinomial: analysis of mark-recapture data with misidentification. *Biometrics*, **66**, 178–185.

McClintock, B.T., Bailey, L.L., Dreher, B.P. & Link, W.A. (2014) Probit models for capture-recapture data subject to imperfect detection, individual heterogeneity and misidentification. *The Annals of Applied Statistics*, **8**, 2461–2484.

McClintock, B.T., Conn, P.B., Alonso, R.S. & Crooks, K.R. (2013) Integrated mod-

eling of bilateral photo-identification data in mark-recapture analyses. *Ecology*, **94**, 1464–1471.
